# ﻿The identity of *Sasaoblongula* C.H.Hu (Poaceae, Bambusoideae, Arundinarieae): evidence from morphology and molecular data

**DOI:** 10.3897/phytokeys.226.101221

**Published:** 2023-05-09

**Authors:** Xing Li, Jing-Bo Ni, Nian-He Xia

**Affiliations:** 1 Key Laboratory of Plant Resources Conservation and Sustainable Utilization & Guangdong Provincial Key Laboratory of Applied Botany, South China Botanical Garden, Chinese Academy of Sciences, CN-510650, Guangzhou, China South China Botanical Garden, Chinese Academy of Sciences Guangzhou China; 2 South China National Botanical Garden, CN-510650, Guangzhou, China South China National Botanical Garden Guangzhou China; 3 University of Chinese Academy of Sciences, CN-100049, Beijing, China University of Chinese Academy of Sciences Beijing China

**Keywords:** bamboo, China, phylogeny, *Pseudosasa*, taxonomy

## Abstract

*Sasaoblongula* was described in 1987 based on a cultivated plant at the bamboo garden of Sun Yat-sen University. This species has two or three branches at the upper nodes, which differ from the rest of *Sasa* species that have a single branch per node. During the field trip to Baishi Town, Yunfu City, Guangdong Province in July 2021, one bamboo species with oblong foliage leaves was collected and matches the isotype. Then, our question was to test the identity of *S.oblongula* concerning other *Sasa* species based on morphology and molecular data. To do that, we sequenced the whole chloroplast genome of *S.oblongula* and did a phylogenetic analysis. Our morphological results indicate that the new collection is *S.oblongula*. The phylogenetic tree showed that *S.oblongula* is close to *Pseudosasa*, instead of *Sasa* species. Therefore, we transferred it to the genus *Pseudosasa*, and a revised description of *P.oblongula* is provided here.

## ﻿Introduction

*Sasaoblongula* C.H.Hu (1987) was described based on two collections, i.e., *Y. L. Yang & C. H. Hu 198001* (Type) and *T. H. Wen & G. Y. Sheng 79413*, from the bamboo garden of Sun Yat-sen University, Guangdong Province. According to its protologue, it was transplanted from somewhere in Guangdong with lack of a detailed address and could be distinguished by having small-medium-sized and oblong foliage leaves. It was well recognized and accepted as a distinctive species in the floras (Hu 1996; Wang and Stapleton 2006; Yi et al. 2008; Xia and Lin 2009; Vorontsova et al. 2016; Shi et al. 2022) and websites like GrassBase-The Online World Grass Flora (Clayton et al. 2016), Tropicos (www.tropicos.org), IPNI (www.ipni.org), POWO (powo.science.kew.org), The Plant List (www.theplantlist.org), GBIF (www.gbif.org). After examining paratype specimen *T. H. Wen & G. Y. Sheng 79413* in N and a failed attempt of searching for it in the bamboo garden of Sun Yat-sen University during the revision of Sasinae Keng f. (Keng 1982), Li (2009) treated it as a suspicious species. Significantly, the type specimens and protologue all demonstrated that this bamboo species possessed two or three branches at upper culm nodes, which conflicted with the strictly solitary branch of *Sasa* at each node (Makino and Shibata 1901; Suzuki 1978; Kobayashi 2017; Qin 2019). Thus, *S.oblongula* should not belong to the genus *Sasa* and may be a member of *Pseudosasa* based on the evidence available.

However, the previous molecular phylogenetic analysis (Zeng et al. 2010) showed a surprising result, namely that *S.oblongula*, eight Japanese *Sasa* species (including generic type), and one *Sasaella*Makino (1929) species, formed a subclade within the Arundinaria clade. To date, most Chinese *Sasa* species except Sasasubg.Sasamorpha have been transferred to *Sinosasa* L.C.Chia ex N.H.Xia, Q.M.Qin & Y.H.Tong and *Yushania* Keng f. (Keng 1957; Qin 2019; Qin et al. 2021; Li et al. 2023). We think that the voucher specimen *Zeng & Zhang 06055* of *S.oblongula* used by Zeng et al. for the molecular analysis is probably misidentified. Thus, the phylogenetic position of *S.oblongula* needs to be further studied with correct samples.

During the field trip to Baishi Town, Yunfu City, Guangdong Province in July 2021, one bamboo species with oblong foliage leaves was found. It matched the isotype very well and shares the same key characters, such as the slightly prominent culm supranodal ridge, the white powdery infranodal region, the glabrous internodes with three branches at an upper node, the solitary secondary branch, three to six foliage leaves clustered at the top of ultimate branches, the small-medium-sized and oblong foliage leaves with glabrous blades and conspicuous transverse veins. Therefore, we are certain that the specimens we collected are *S.oblongula*. Then, our question was to test the identity of *Sasaoblongula* concerning other *Sasa* species based on morphology and molecular data.

## ﻿Materials and methods

### ﻿Morphology

The sample of *Sasaoblongula* was collected from Hengjing Villiage, Baishi Town, Yunfu City, Guangdong, China during a field trip in July 2021. Observations and measurements were taken using a magnifier (SZ-6) and a ruler with a scale of 0.5 mm. Some minor characters such as indumentum on ligules of both culm leaves and foliage leaves were observed with a stereomicroscope (Mshot MZ101). The description was made based on both living and dried material as well as relevant literature (e.g. Hu 1987, 1996; Wang and Stapleton 2006; Xia and Lin 2009). Comparisons between *S.oblongula* and *Pseudosasacantorii* were conducted based on protologue and type specimens, and relevant specimens involved in the protologue of *Arundinariacantorii* (≡*Pseudosasacantorii*). The descriptive terms follow Beentje (2016) and herbaria acronyms follow Thiers (2021).

### ﻿Sampling

For obtaining reliable results, a reasonable proof strategy with two steps was designed to identify the systematic position of *S.oblongula*. The first step is to test whether *S.oblongula* belongs to *Sasa* based on our plastid tree. The second step is to identify which genus *S.oblongula* belongs to based on SNP tree, mainly due to low discrimination rates for those ‘three-branched’ genera in plastid results (Guo et al. 2021). For the plastid tree, a total of 24 species from 11 genera were sampled. *Bambusamultiplex* and *Dendrocalamusstrictus* were set as the outgroups. All accession numbers and voucher information are listed in Table 1. For the SNP tree, a total of 14 species from seven genera belonging to subtribe Arundinarieae were included. *Chimonobambusasangzhiensis* was set as outgroup. Particular emphasis in our taxon sampling was placed on the inclusion that several key generic types were all involved in this study, including *Acidosasa*, *Indosasa*, *Oligostachyum*, *Pseudosasa*, and *Sasa*.

### ﻿DNA extraction and sequencing

Young leaves at the vegetative growth stage were collected in the field. Total genomic DNA was isolated from silica-dried leaves following the manufacturer’s specifications TIANGEN Genomic DNA Extraction Kit (TIANGEN, Beijing, China). DNA samples of concentration up to standard (≥1 μg) were sheared into fragments using Covaris M220 (Covaris, Woburn, MA). Insert size of 350 bp fragments were enriched by PCR, and the paired-end (2 × 150 bp) libraries were constructed on NovaSeq 6000 platform. About 20G deep genome skimming (DGS) data were generated. Finally, adapters and low-quality reads were filtered from raw data using Fastp v 0.23.1 (Chen et al. 2018) software.

### ﻿Plastome assembly and chloroplast DNA regions mapping

The filtered clean reads were utilized to de novo assemble complete chloroplast (cp) genomes using GetOrganelle v 1.6.2 pipeline (Jin et al. 2018). Six k-mer values, including 21, 45, 65, 85, 105,125, were set for plastid contigs connection. Subsequently, the filtered plastid reads were transferred to Bandage (Wick et al. 2015) software for visualization processing. Two opposite plastid sequences exported from Bandage were aligned with the reference sequence *Phyllostachysedulis* (GenBank accession No. HQ337796), and one that matched the genomic direction of the reference was retained. The final cp genomes were manually corrected in Geneious 9.1.4 (Kearse et al. 2012).

**Table 1.** List of 24 species with species names, voucher information and GenBank accession numbers for the plastid tree based on eight combined plastid sequences extracted from whole chloroplast genomes (WCG).

Taxon	Voucher information	GenBank accession								
		WCG	atpI/H	psaA-ORF170	rpl32-trnL	rpoB-trnC	rps16-trnQ	trnD/T	trnS/G	trnT/L
**Ingroup**										
*Acidosasapurpurea* (Hsueh & Yi) Keng f.	Zhang 07067 (KUN)	/	GU355020	GU355340	GU355500	GU354382	GU354540	GU354700	GU355180	GU354860
*Fargesiaedulis* Hsueh & Yi	Li & Zhang 07051 (KUN)	/	GU355130	GU355450	GU355610	GU354490	GU354650	GU354810	GU355290	GU354970
*Indocalamussinicu*s (Hance) Nakai	Zeng & Zhang 06081 (KUN)	/	GU355153	GU355473	GU355633	GU354513	GU354673	GU354833	GU355313	GU354993
*Pseudosasahindsii* (Munro) C.D.Chu & C.S.Chao	Zhang 07013 (KUN)	/	GU355030	GU355350	GU355510	GU354392	GU354550	GU354710	GU355190	GU354870
*Pseudosasalongiligula* Wen	Zhang 07021 (KUN)	/	GU355067	GU355387	GU355547	GU354429	GU354587	GU354747	GU355227	GU354907
*Sasakurilensis* (Ruprecht) Makino et Shibata	Triplett 223 (KUN)	/	GU355137	GU355457	GU355617	GU354497	GU354657	GU354817	GU355297	GU354977
*Sasaoblongula* C.H.Hu	Zeng & Zhang 06055 (KUN)	/	GU355112	GU355432	GU355592	GU354472	GU354632	GU354792	GU355272	GU354952
*Sasapalmata* (Mitford) Camus	Triplett 228 (KUN)	/	GU355141	GU355461	GU355621	GU354501	GU354661	GU354821	GU355301	GU354981
*Sasasenanensis* (Franchet & Savatier) Rehder	Triplett 146 (KUN)	/	GU355111	GU355431	GU355591	GU354471	GU354631	GU354791	GU355271	GU354951
*Sasatsuboiana* Makino	Triplett 133 (KUN)	/	GU355139	GU355459	GU355619	GU354499	GU354659	GU354819	GU355299	GU354979
*Sasaveitchii* (Carriere) Rehder	Triplett 126 (KUN)	/	GU355138	GU355458	GU355618	GU354498	GU354658	GU354818	GU355298	GU354978
*Yushaniamaculat*a Yi	Zhang 08006 (KUN)	/	GU355084	GU355404	GU355564	GU354444	GU354604	GU354764	GU355244	GU354924
*Acidosasagaluca* B.M.Yang	CZY56 (IBSC)	OP850353								
*Pseudosasaoblongula* (C.H.Hu) N. H. Xia & X. Li	XNH187 (IBSC)	OP874594	/	/	/	/	/	/	/	/
*Pseudosasajaponica* (Sieb. & Zucc. ex Steu.) Maki. ex Naka.	NH028 (IBSC)	OP874595	/	/	/	/	/	/	/	/
*Pseudosasaamabilis* (McClure) P. C. Keng ex S. L. Chen et al.	NH032 (IBSC)	OP850358	/	/	/	/	/	/	/	/
*Indosasacrassiflora* McClure	BH58 (IBSC)	OK558536	/	/	/	/	/	/	/	/
*Sinosasafanjingshanensis* N.H.Xia, Q.M.Qin & J.B.Ni	BH124 (IBSC)	OP850348	/	/	/	/	/	/	/	/
*Sinosasalongiligulata* (McClure) N.H.Xia, Q.M.Qin & J.B.Ni	CZY163 (IBSC)	OP850351	/	/	/	/	/	/	/	/
*Oligostachyumsulcatum* Z.P.Wang & G.H.Ye	Not provided by the author	MW190089	/	/	/	/	/			
*Indosasasinica* C.D.Chu & C.S.Chao	MPF10034 (KUN)	JX513422	/	/	/	/	/	/	/	/
*Oligostachyumshiuyingianum* (Chia & But) Wang et Ye	DZL09122 (KUN)	JX513423	/	/	/	/	/	/	/	/
**Outgroup**										
*Bambusamultiplex* (Loureiro) Raeu. ex Schu. & J. H. Schu.	Not provided by the author	KJ722536	/	/	/	/	/	/	/	/
*Dendrocalamusstrictus* (Roxburgh) Nees	zmy018 (KUN)	MK679802								

After referring to previous plastid phylogeny studies of Arundinarieae (Zeng et al. 2010; Zhang et al. 2012), eight plastid DNA regions (*atpI*-*atpH*, *psaA*-*ORF170*, *rpl32*-*trn*L, *rpoB*-*trnC*, *rps16*-*trnQ*, *trnD*-*trnT*, *trnS*-*trnG* and *trnT*-*trnL*) were selected to reconstruct plastid phylogenetic tree. Our cp genomes were annotated from eight DNA regions of *Acidosasapurpurea* with ≥ 70% sequence similarity in Geneious. Then, all the annotated plastid DNA regions were extracted from whole cp genomes. Sequence directions were visualized and adjusted using Mauve v 2.4.0 (Darling et al. 2004).

### ﻿SNP calling

The latest high-quality genome sequence of moso bamboo (*Phyllostachysedulis*) (Zhao et al. 2018) was selected as the chromosome-level reference genome to build an index using the software SAMtools v 1.9 (Danecek et al. 2021) and Picard v 2.27.3 (Broad Institute 2019). After filtration of low-quality data, our clean reads were processed in removal of duplicates using Fastuniq v 1.1 (Xu et al. 2012). New filtered paired reads were aligned to the reference genome by Bowtie2 v 2.4.4 (Langmead and Salzberg 2012) with the parameter of minimum acceptable alignment score for L, 0.3, 0.3. After that, SAMtools was further employed to sort alignment (BAM files). Picard was utilized to remove duplicates again with the parameter “MarkDuplicates”. GATK v 4.2.2.0 (Van der Auwera and O’Connor 2020) was performed to anchor variant calling including SNP and InDel using the joint calling method “HaplotypeCaller” in the genomic variant call format (GVCF). Each sample based on reads with mapping quality was set as at least 10 and the kmer size was set as 10 to 25. After completion of variants calling, the tool “CombineGVCFs” in GATK was carried out to combine all the GVCF files. The tool “GenotypeGVCFs” was then utilized to identify joint-called variants. Subsequently, the tool “SelectVariants” was implemented to select single nucleotide polymorphic sites (SNPs). Filtration of SNPs of low quality was then conducted in the tool “VariantFiltration” with the parameter “QD < 2.0, MQ < 40.0, FS > 60.0, SOR > 3.0, MQRankSum < -12.5 and ReadPosRankSum < -8.0”. Finally, the tool “SelectVariants” was run to extract filtered SNPs.

For a reliable phylogenetic tree based on SNP dataset, we considered that filtered raw SNPs with high missing genotype rates and low minor allele frequency will affect the accuracy of the phylogenetic trees and thus should be removed. Therefore, plink v 1.90b4.6 (Purcell et al. 2007) was operated to filter those low-quality SNPs with parameter “geno” set as 0.1 and “maf” set as 0.01. Filtered variants were then pruned with the parameter “indep-pairwise” set as 50, 10, 0.2, representing its window size, a variant count to shift the window and pairwise r2 threshold for SNPs, respectively. Finally, new clean SNP dataset was generated, and the GVCF format was transferred to PHYLIP format for phylogenetic analysis using the python script “vcf2phylip.py” (Ortiz 2019).

### ﻿Alignments construction and phylogenetic trees inference

Chloroplast DNA regions and SNP dataset were utilized to reconstruct the phylogenetic tree, respectively. Eight plastid matrices were aligned using MAFFT v 7.450 (Katoh and Standley 2013) and concatenated as a super matrix. Maximum likelihood (ML) tree was inferred for plastid and SNP datasets using IQTREE v 1.6 in SH-aLRT test and ultrafast bootstrap (UFBoot) value (Nguyen et al. 2015). Node supports rates with SH-aLRT ≥ 80% and UFboot ≥ 95% were reliable and shown on each node. The final results were visualized with Figtree 1.4.4 (Rambaut 2018).

## ﻿Result

### ﻿Morphological comparison

*Ssasaoblongula* has leptomorph rhizome, glabrous culm internodes, white powdery infranodal region, flat or slightly prominent nodes and culm supranodal ridge, mostly solitary branch at lower culm nodes and two to three (Fig. 3E, if three branches, central slightly dominant than lateral) branches at mid and upper culm nodes, glabrous culm leaf sheath (Fig. 3G) with erect and lanceolate blades, falcate auricles and ligules with ciliolate margin, glabrous foliage leaves blades and conspicuous transverse veins. These vegetative characters mentioned above make it fit well with the circumscription of *Pseudosasa* Makino ex Nakai (1925), rather *Sasa*. After examining the specimens of similar species and referring to the related literature (Munro 1868; Chia et al. 1983), we found that *S.oblongula* is most similar to *P.cantorii* (Munro) P. C. Keng ex S. L. Chen et al. (Zhu et al. 2006) by sharing one to three branches per nodes, glabrous internodes, the white powdery infranodal region, slightly prominent supranodal ridge, culm leaf sheath with falcate auricles, erect and lanceolate blades with serrulate margin, foliage leaf sheath with ciliate margin and truncate ligules, glabrous foliage leaf blades with conspicuous transverse veins, but differs by having nearly solid (vs. hollow) culm internode with appressed (vs. patent) branches, intravaginal (vs. transferred) and glabrous (vs. setose) abaxially culm leaf sheath with ciliate upper (vs. wholly) margin and arched (vs. truncate) ligules with ciliolate (vs. glabrous) margin, 3–6 foliage leaves with irregular (vs. coplanar) arrangement clustered at the top ultimate branch, glabrous (vs. hirsute) abaxially foliage leaf sheath with 1–4 mm (vs. 5–13 mm) long length per adjacent sheath apex, small-medium-sized (7–10 × 1.5–2.6 cm vs. 12.5–25 × 2.5–3.2 cm) foliage leaf blades with 6–7-paired (vs. 7–9-paired) secondary veins. A more detailed comparison between the two species is provided in Table 2.

### ﻿Phylogenetic analysis

To make clear the position of *S.oblongula* and its relationship with *P.cantorii*, phylogenetic Maximum likelihood analysis was conducted based on plastid, and SNP dataset was shown with SH-aLRT and UFboot values noted at each node. Our plastid phylogenetic tree indicated that *S.oblongula* was distantly related to those Japanese *Sasa* species (including generic type) (Fig. 4, SH-aLRT=99.5% & UFboot=100%) and those *Sinosasa* species (SH-aLRT=100% & UFboot=100%) previously assigned to *Sasa* from China (Qin et al. 2021). The SNP phylogenetic tree suggested that *S.oblongula* was sister to *P.cantorii* with strong support (Fig. 5, SH-aLRT=100% & UFboot=100%), supporting our morphological study well.

**Table 2.** Comparison of *Pseudosasaoblongula* and *Pseudosasacantorii*.

Morphology	*Pseudosasaoblongula*	*Pseudosasacantorii*
**Culm internode**	Nearly solid, with appressed branches	Hollow, with patent branches
**Culm leave**s	Intravaginal, glabrous abaxially	Transferrd, setose abaxially and readily deciduous when old
**Margin**	Ciliate on the upper	Ciliate wholly
**Ligule**	Arched, with ciliolate margin	Truncate, with glabrous margin
**Foliage leaves**	3–6, with irregular arrangement clustered at the top ultimate branch	4–7, with a coplanar arrangement at the top ultimate branch
**Sheath**	Glabrous abaxially, 1–4 mm long per adjacent sheath apex	Hirsute abaxially, 5–13 mm long per adjacent sheath apex
**Blades**	7–10 × 1.5–2.6 cm, with 6–7-paired secondary veins	12.5–25 × 2.5–3.2 cm, with 7–9-paired secondary veins

## ﻿Taxonomic treatment

### ﻿ Pseudosasa oblongula

Taxon classificationPlantaePoalesPoaceae

(C.H.Hu) N.H.Xia & X.Li comb. nov.

A5A483B5-9430-53B4-9589-B7491F986C2F

urn:lsid:ipni.org:names:77317186-1

Figs 1 , 2 , 3

Sasa oblongula C. H. Hu, J. Bamboo Res., 6(4):18 (1987). Basionym.

#### Type.

**Figure 1.**
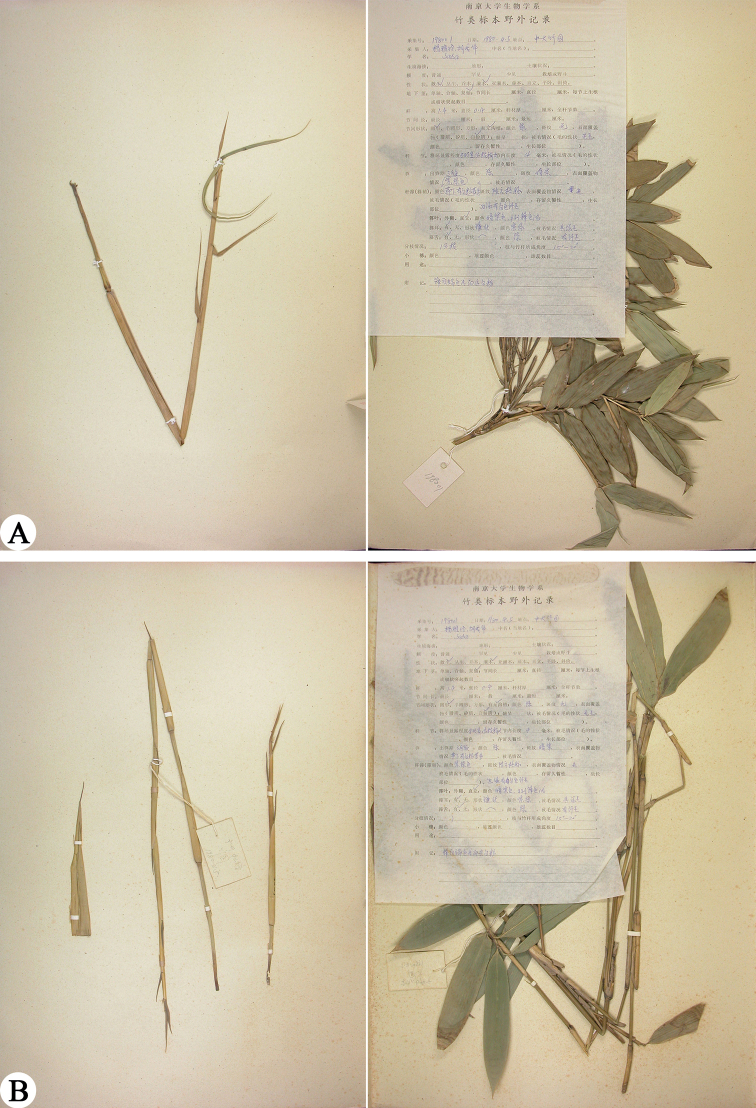
Isotypes of *Pseudosasaoblongula, Y.L.Yang & C.H.Hu 198001* (**A** N019023154 **B** N019023156). Photos downloaded from Chinese Virtual Herbarium (https://www.cvh.ac.cn/).

China. Guangdong: type locality unknown, cultivated in Bamboo Garden, Sun Yat-sen University, 5 April 1980, *Y. L. Yang & C. H. Hu 198001* (holotype: N, photo!; isotypes: N019023154, Fig. 1A; N019023155, image!; N019023156, Fig. 1B)

#### Description.

Shrubby bamboo, rhizomes leptomorph. Culm erect, 1–1.5 m tall, 2–7 cm in diameter; branches appressed, usually 1 branch at the lower culm nodes and 2–3 at the mid or upper nodes of culm (if 3 branches, central slightly dominant than lateral); internodes terete or slightly flattened above branches, 5–27 cm long, glabrous, nearly solid; nodes flat or slightly prominent, white powdery under nodes; supranodal ridge flat or slightly prominent; intranodes 5–8 mm high, glabrous; culm buds solitary, ovate to elliptic, light yellow, puberulent abaxially at upper part, ciliolate on the upper margin, apex obtuse. Culm leaf sheath persistent or deciduous later, intravaginal, thinly leathery, 1/3–2/3 as long as internodes, glabrous abaxially, ciliate on the upper margin and sometimes glabrescent, longitudinal ribs conspicuous; sheath scar with remains of sheath base; auricles falcate to long-elliptic, obliquely ascending, 2–4 × 1–2 mm; oral setae erect or slightly curved, 3–10 mm long; ligule entire, 0.5–1 mm high, asperous abaxially, ciliolate on the margin, apex arched; blades narrowly lanceolate to lanceolate, erect, glabrous abaxially, margin sparsely serrate. Foliage leaves 3–6 clustered at the top of ultimate branches, with irregular arrangement; sheath thinly leathery, glabrous abaxially, margins densely ciliate, sometimes glabrous, thinly white-powdery, longitudinal ribs conspicuous, length per adjacent sheath apex very short, 1–4 mm; auricles undeveloped, ovate to falcate or absent, 1–3 × 1–1.5 mm; oral setae erect or curved, 5–10 mm long, usually deciduous when old; inner ligule 0.5–1 mm high, densely ciliolate on margin, apex truncate; outer ligule ca. 0.5 mm high, ciliolate on margin; blades oblong to oblong-lanceolate, papyraceous, 7–10 × 1.5–2.6 cm, glabrous adaxially and abaxially, apex acute to attenuate, base obtuse to unequal rounded, margin serrate; secondary veins 6–7 pairs, tertiary veins 6–7 pairs, transverse veins conspicuous; petioles 2–4 mm long, glabrous; Inflorescence unknown.

**Figure 2.**
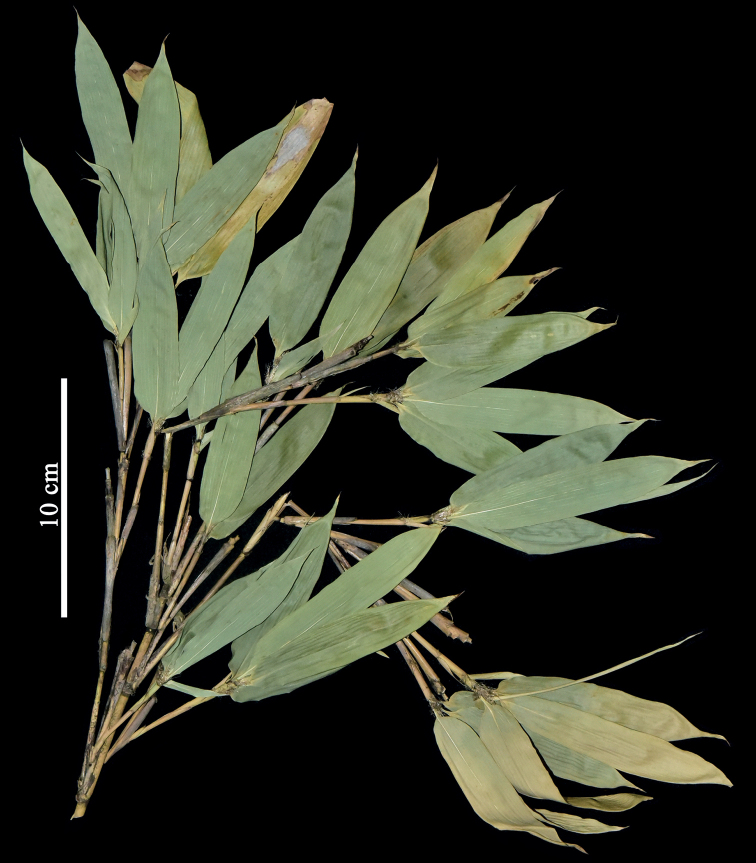
*Pseudosasaoblongula* (C.H.Hu) N. H. Xia & X. Li, *N. H. Xia XNH-187* (IBSC). Photo by Xing Li.

**Figure 3.**
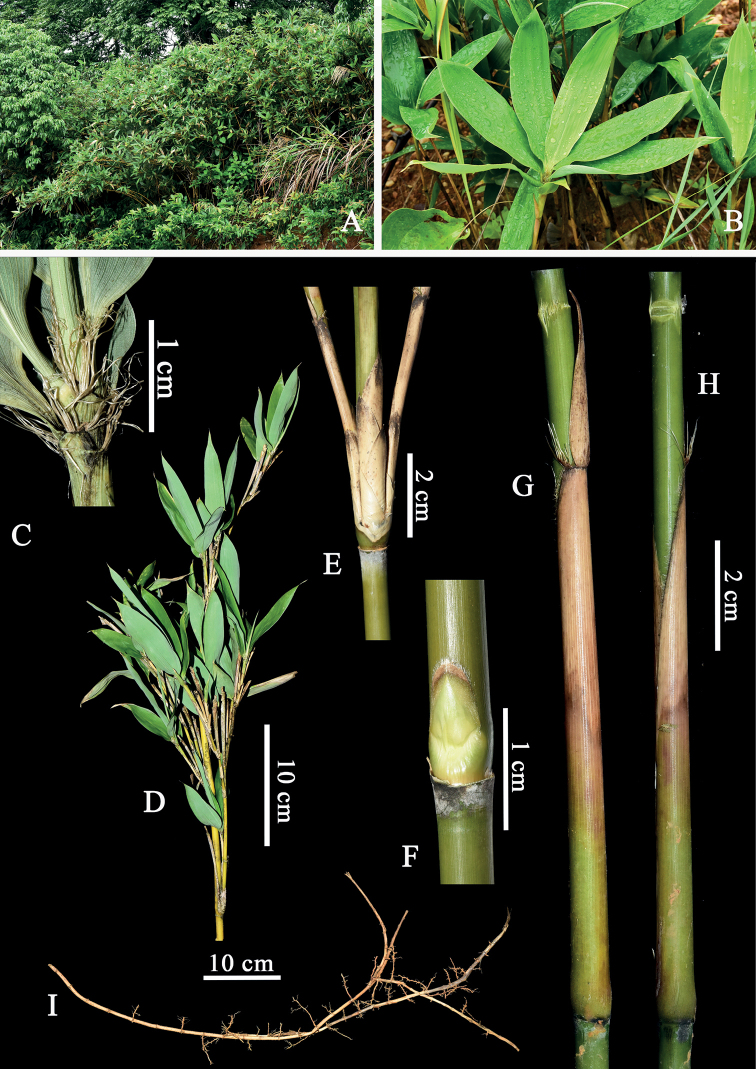
*Pseudosasaoblongula* (C.H.Hu) N. H. Xia & X. Li **A** habit **B** foliage leaf, showing oblong blade **C** part of terminal branch, showing sheath and ligule **D** partial culm and branches **E** nodes of the upper culm, showing three branches complement **F** culm bud **G, H** culm leaf, showing lanceolate blades, falcate auricles and glabrous sheath **I** leptomorph rhizomes. Photos **E, F** by Zhuo-yu Cai, others by Xing Li.

#### Phenology.

New shoots were produced from March to July.

#### Vernacular names.

Jǔ Yè Shǐ Zhú (Chinese pronunciation); 矩叶矢竹 (Chinese name).

#### Additional specimens examined.

***Pseudosasaoblongula***: China. Guangdong: type locality unknown, cultivated in Bamboo Garden, Sun Yat-sen University, 12 April 1979, *T. H. Wen & G. Y. Sheng 79413* (JSB518673 image!); Yunfu City, Yunan County, Baishi Town, Hengjing village, 4 July 2021, *N. H. Xia XNH-187* (IBSC!); ibid. 22°52'8"N, 111°51'59"E, elev. 206 m, 11 June 2022, *J. B. Ni & X. Li LX142* (IBSC!). ***Pseudosasacantorii***: China. Hong Kong: Lantau Island, *Cantor s.n.*, quoad foliage leaf (K000876243, image!); Green Island, 1 May 1981, *L. C. Chia et al. Nan-zhu 2875* (US 00031256, image!); Shatin, Siu Lek Yuen, 18 Oct. 1980, *L. C. Chia et al. Nan-zhu 2823* (US00031257, image!); ibid. *L. C. Chia et al. Nan-zhu 2830* (US00031259, image!); Xinjie, Jiadaoli Farm, 22 April 1981, *Nan-zhu 2867* (IBSC!); ibid. 15 October 1980, *Nan-zhu 2810* (IBSC!).

## ﻿Discussion

**Figure 4.**
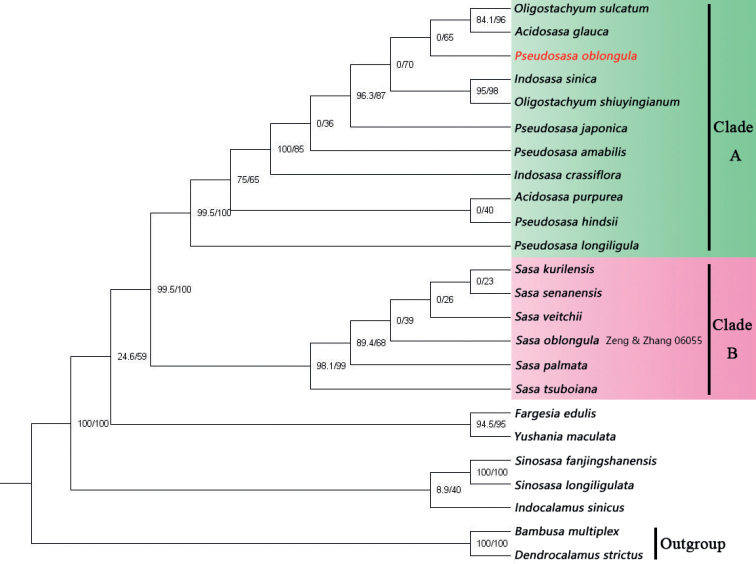
Phylogenetic relationships of *Pseudosasaoblongula* and other 23 species of which most taxa belong to the tribe Arundinarieae based on eight combined plastid sequences. The value of the SH-aLRT test (left) and ultrafast bootstrap (right) are indicated on each node.

**Figure 5.**
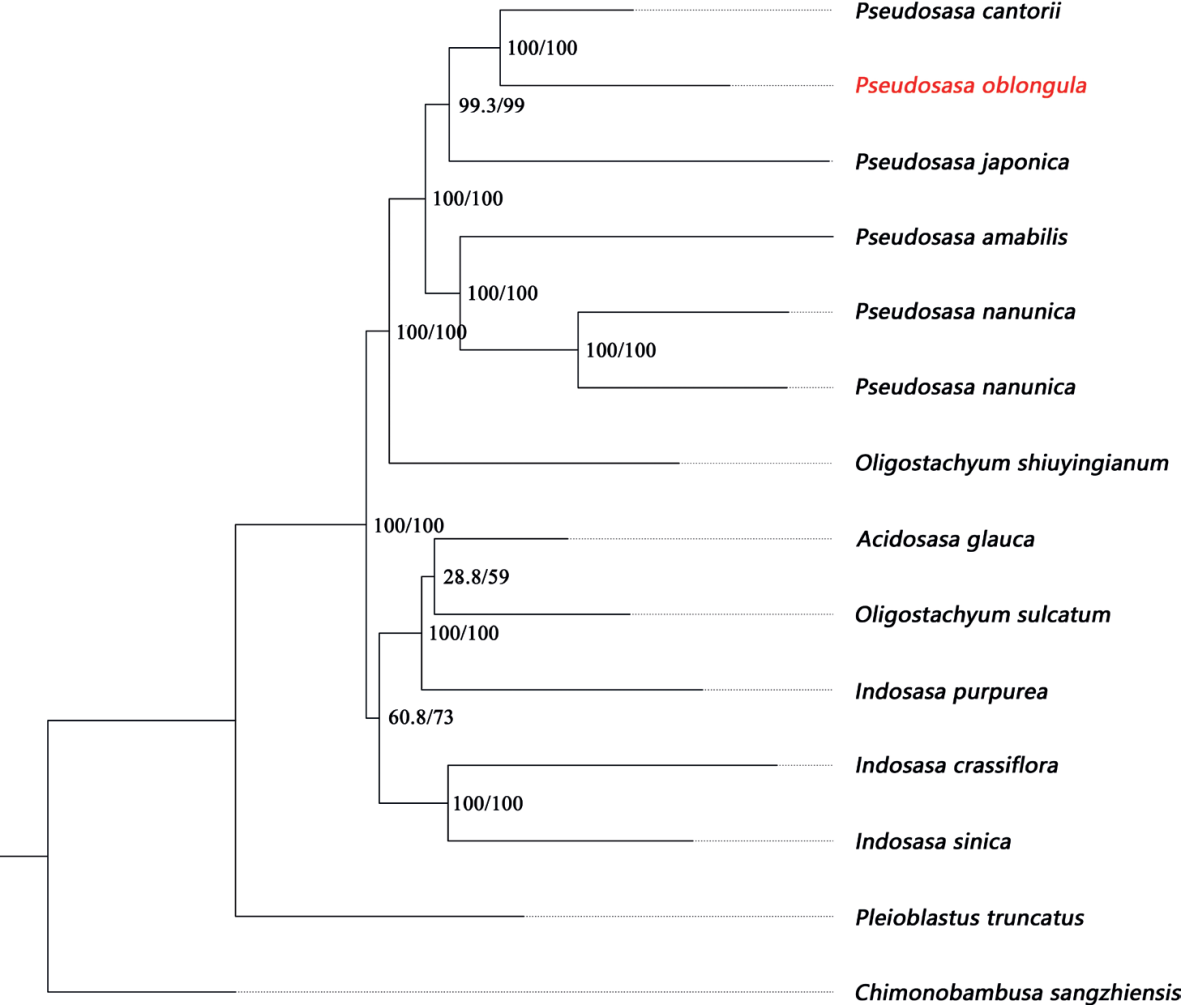
Phylogenetic relationships of *Pseudosasaoblongula* and other 13 species belong to the tribe Arundinarieae based on single nucleotide polymorphism dataset. The value of SH-aLRT test (left) and ultrafast bootstrap (right) are indicated on each node.

*Sasaoblongula*, mainly characterized by its oblong foliage leaves, was published based on sterile materials introduced in the bamboo garden of Sun Yat-sen University. It differed from Japanese *Sasa* species by having 1–3 branches per node (vs. 1 branch) and remote geographic distribution, indicating that it was not obviously the member of *Sasa*. After the examination of the voucher specimen *Zeng & Zhang 06055* from Zeng et al. (2010), we were certain that this specimen does not represent *S.oblongula* since it possesses solitary branch at upper culm nodes, undeveloped or absent culm leaf auricles, and long-lanceolate foliage leaf blades. Our phylogenetic study revealed that the actual *S.oblongula* and those Japanese *Sasa* species are dispersed in two different clades (Fig. 4, Clade A & B). Furthermore, it and *P.cantorii* form a well-supported clade with two different branch lengths based on SNP phylogenetic tree (Fig. 5), supporting the result of morphology.

However, previous studies (Zhang et al. 2012; Guo et al. 2021) showed that *Pseudosasa* is polyphyletic, and the phylogenetic relationships between *Pseudosasa* and several other genera of subtribe Arundinariinae (Zhang et al. 2020), such as *Pleioblastus*, *Oligostachyum*, *Indosasa*, etc., have not been resolved. *Sasaoblongula* was closely related to Chinese *Pseudosasa* species in morphology and phylogeny, and thus was congruently assigned to the genus *Pseudosasa* here. Accordingly, a new combination *Pseudosasaoblongula* (C. H. Hu) N. H. Xia & X. Li was proposed.

## Supplementary Material

XML Treatment for Pseudosasa oblongula

## Supplementary materials

Supplementary material 1 SNP matrix Data type phylogenetic Explanation note This SNP matrix contains 14 species with 36490 bp. https://binary.pensoft.net/file/847652This dataset is made available under the Open Database License (http://opendatacommons.org/licenses/odbl/1.0/). The Open Database License (ODbL) is a license agreement intended to allow users to freely share, modify, and use this Dataset while maintaining this same freedom for others, provided that the original source and author(s) are credited.Xing Li

## References

[B1] Beentje H (2016) The Kew Plant Glossary, Second Edition. Royal Botanic Gardens, Kew, London, 184 pp.

[B2] Broad Institute (2019) Picard Toolkit. Broad Institute, GitHub repository.

[B3] Chen SF Zhou YQ Chen YR Gu J (2018) fastp: An ultra-fast all-in-one FASTQ preprocessor. Bioinformatics 34(17): i884–i890. 10.1093/bioinformatics/bty560 PMC6129281 30423086

[B4] Chia LZ Fung HL Paul PB (1983) Notes on Gramineae: Bambusoideae in Hong Kong. Kew Bulletin 37(4): e591. 10.2307/4109728

[B5] Clayton WD Vorontsova M Harman KT Williamson H (2016) GrassBase-The online world grass flora. https://kew.org/data/grasses-db/index.html [accessed 19 March 2023]

[B6] Danecek P Bonfield JK Liddle J Marshall J Ohan V Pollard MO Whitwham A Keane T McCarthy SA Davies RM Li H (2021) Twelve years of SAMtools and BCFtools. GigaScience 10(2): giab008. 10.1093/gigascience/giab008 PMC7931819 33590861

[B7] Darling ACE Mau B Blattner FR Perna NT (2004) Mauve: Multiple alignment of conserved genomic sequence with rearrangements. Genome Research 14(7): 1394–1403. 10.1101/gr.2289704 15231754 PMC442156

[B8] Guo C Ma PF Yang GQ Ye XY Guo Y Liu JX Liu YL Eaton DAR Guo ZH Li DZ (2021) Parallel ddRAD and genome skimming analyses reveal a radiative and reticulate evolutionary history of the temperate bamboos. Systematic Biology 70(4): 756–773. 10.1093/sysbio/syaa076 33057686 PMC8208805

[B9] Hu CH (1987) A new species of *Sasa* from Guangdong. Journal of Bamboo Research 6(4): 18–20.

[B10] Hu CH (1996) *Sasa* Makino et Shibata. In: Geng BJ Wang ZP (Eds) Flora Reipublicae Popularis Sinicae (Vol.9). Science Press, Beijing, 662–675.

[B11] Jin JJ Yu WB Yang JB Song Y Yi TS Li DZ (2018) GetOrganelle: a simple and fast pipeline for de novo assembly of a complete circular chloroplast genome using genome skimming data. bioRxiv 256479. 10.1101/256479

[B12] Katoh K Standley DM (2013) MAFFT: multiple sequence alignment software version 7: improvements in performance and usability. Molecular Biology and Evolution 30(4): 772–780. 10.1093/molbev/mst010 23329690 PMC3603318

[B13] Kearse M Moir R Wilson A Stones-Havas S Cheung M Sturrock S Buxton S Cooper A Markowitz S Duran C Tierer T Ashton B Meintjes P Drummond A (2012) Geneious Basic: An integrated and extendable desktop software platform for the organization and analysis of sequence data. Bioinformatics 28(12): 1647–1649. 10.1093/bioinformatics/bts199 22543367 PMC3371832

[B14] Keng PC (1957) One new genus and two new species of Chinese bamboos. Zhiwu Fenlei Xuebao 6: 355–362.

[B15] Keng PC (1982) A revision of the genera of bamboos from the world. Journal of Bamboo research 1(1): 1–19.

[B16] Kobayashi M (2017) The Illustrated Book of Plant Systematics in Color Bambusoideae in Japan. The Hokuryukan Press, 435 pp.

[B17] Langmead B Salzberg SL (2012) Fast gapped-read alignment with Bowtie 2. Nature Methods 9(4): 357–359. 10.1038/nmeth.1923 22388286 PMC3322381

[B18] Li L (2009) Systematic Studies on Sasinae. PhD Thesis, Nanjing Forestry University, China.

[B19] Li X Ni JB Tan F Tong YH Xia NH (2023) *Yushaniatomentosa* (Poaceae, Bambusoideae), a new combination from Guangxi. PhytoKeys 218: 11–18. 10.3897/phytokeys.218.97312 36762274 PMC9846283

[B20] Makino T (1929) A Contribution to the Knowledge of the Flora of Japan. Shokubutsu Kenkyu Zasshi 6: 1–15.

[B21] Makino T Shibata K (1901) On *Sasa*, a new genus of Bambuseae, and its affinities. Botanical Magazine Tokyo 15(168): 18–31. 10.15281/jplantres1887.15.168_18

[B22] Munro W (1868) A Monograph of the Bambusaceae, including Description of all the Species. Transactions of the Linnean Society of London 26(1): e111. 10.1111/j.1096-3642.1968.tb00502.x

[B23] Nakai T (1925) Two new genera of Bambusaceae, with special remarks on the related genera growing in eastern Asia. Journal of the Arnold Arboretum 6: e150. 10.5962/bhl.part.24130

[B24] Nguyen LT Schmidt HA von Haeseler A Minh BQ (2015) IQ-TREE: A fast and effective stochastic algorithm for estimating maximum likelihood phylogenies. Molecular Biology and Evolution 32(1): 268–274. 10.1093/molbev/msu300 25371430 PMC4271533

[B25] Ortiz EM (2019) vcf2phylip v2.0: convert a VCF matrix into several matrix formats for phylogenetic analysis.

[B26] Purcell S Neale B Todd-Brown K Thomas L Ferreira MAR Bender D Maller J Sklar P de Bakker PIW Daly MJ Sham PC (2007) PLINK: A toolset for whole-genome association and population-based linkage analysis. American Journal of Human Genetics 81(3): 559–575. 10.1086/519795 17701901 PMC1950838

[B27] Qin QM (2019) Taxonomic studies of *Sasa* Makino & Shibata and *Gigantochloa* Kurz ex Munro (Poaceae: Bambusoideae) from China. PhD Thesis, University of Chinese Academy of Sciences, China.

[B28] Qin QM Tong YH Zheng XR Ni JB Xia NH (2021) *Sinosasa* (Poaceae: Bambusoideae), a new genus from China. Taxon 70(1): 27–47. 10.1002/tax.12422

[B29] Rambaut A (2018) FigTree, version v.1.4.4. http://tree.bio.ed.ac.uk/software/figtree/ [accessed 25 October 2020]

[B30] Shi JY Che BY Zhang YX Zhou DQ Ma LS Yao J (2022) *Sasa* Makino & Shibata. In: Yi TP Shi JY Zhang YX Zhou DQ Ma LS Yao J (Eds) Illustrated Flora of Bambusoideae in China (Vol.2). Science Press, Beijing & Springer Nature, Singapore, 381–396. 10.1007/978-981-16-2758-3_43

[B31] Suzuki S (1978) Index to Japanese Bambusaceae. Gakken Press, 384 pp.

[B32] Thiers B (2021) Index Herbariorum: A global directory of public herbaria and associated staff. New York Botanical Garden’s Virtual Herbarium. http://sweetgum.nybg.org/ih

[B33] Van der Auwera GA O’Connor BD (2020) Genomics in the Cloud: Using Docker, GATK, and WDL in Terra (1^st^ edn). O’Reilly Media, 506 pp.

[B34] Vorontsova MS Clark LG Dransfield J Govaerts R Baker WJ (2016) World Checklist of Bamboos and Rattans. INBAR Technical Report No. 37. International Network of Bamboo & Rattan, Beijing, 454 pp.

[B35] Wang ZP Stapleton CMA (2006) *Sasa* Makino et Shibata. In: Wu ZY Raven P (Eds) Flora of China (Vol.22). Science Press, Beijing & Missouri Botanical Garden Press, St. Louis, 109–112.

[B36] Wick RR Schultz MB Justin Z Holt KE (2015) Bandage: Interactive visualization of *de novo* genome assemblies. Bioinformatics 31(20): 3350–3352. 10.1093/bioinformatics/btv383 26099265 PMC4595904

[B37] Xia NH Lin RS (2009) Bambusoideae. In: South China Botanical Garden, Chinese Academy of Sciences (Eds) Flora of Guangdong (Vol.9). Guangdong Science and Technology Press, Guangzhou, 224–325.

[B38] Xu H Qian J Pang X Song J Qian G Chen J Chen S (2012) FastUniq: A fast de novo duplicates removal tool for paired short reads. PLoS ONE 7(12): e52249. 10.1371/journal.pone.0052249 PMC3527383 23284954

[B39] Yi TP Shi JY Ma LS Wang HT Yang L (2008) Iconographia Bambusoidearum Sinicarum. Science Press, Beijing, 763 pp.

[B40] Zeng CX Zhang YX Triplettd JK Yang JB Li DZ (2010) Large multi-locus plastid phylogeny of the tribe Arundinarieae (Poaceae: Bambusoideae) reveals ten major lineages and low rate of molecular divergence. Molecular Phylogenetics and Evolution 56(2): 821–839. 10.1016/j.ympev.2010.03.041 20381627

[B41] Zhang YX Zeng CX Li DZ (2012) Complex evolution in Arundinarieae (Poaceae: Bambusoideae): Incongruence between plastid and nuclear GBSSI gene phylogenies. Molecular Phylogenetics and Evolution 63(3): 777–797. 10.1016/j.ympev.2012.02.023 22415014

[B42] Zhang YX Guo C Li DZ (2020) A new subtribal classification of Arundinarieae (Poaceae, Bambusoideae) with the description of a new genus. Plant Diversity 42(3): 127–134. 10.1016/j.pld.2020.03.004 32695944 PMC7361428

[B43] Zhao HS Gao ZM Wang L Wang JL Wang SB Fei BH Chen CH Shi CC Liu XC Zhang HL Lou YF Chen LF Sun HY Zhou XQ Wang SN Zhang C Xu H Li LC Yang YH Wei YL Yang W Gao Q Yang HM Zhao SC Jiang ZH (2018) Chromosome-level reference genome and alternative splicing atlas of moso bamboo (*Phyllostachysedulis*). GigaScience 7(10): giy115. 10.1093/gigascience/giy115 PMC6204424 30202850

[B44] Zhu ZD Li DZ Stapleton CMA (2006) *Pseudosasa* Makino ex Nakai. In: Wu ZY Raven P (Eds) Flora of China (Vol.22). Science Press, Beijing & Missouri Botanical Garden Press, St. Louis, 115–121.

